# Blood and Blood-Pressure

**Published:** 1901-06

**Authors:** 


**Blood and Blood-Pressure.** By GEORGE OLIVER, M.D. Pp. 272. London: H. K. Lewis. 1901.

This work, by a physician, is both a contribution to the physiological side of medicine, and also gives valuable information for the clinical aspect of circulatory disturbances. It is gratifying to see that a physician in active practice is able to add appreciably to our knowledge of the physiology of the circulation. In these days of specialism it is unfortunately not so common an occurrence as is to be desired.

If physiology is to be really useful to the medical student, he should be able to carry on and apply his physiological knowledge when he comes to the bedside; and if there were any class of disorders in which this should *à priori* be most readily done, it would be those of the heart and vessels. But unfortunately this has hitherto only been the case to a very disappointing extent, and it was this aloofness from clinical work and practice of the physiological facts pertaining to the circulation so painfully acquired by the student that led Dr. Oliver to make the successful step that is embodied in the book before us to bring the two into harmony. The result is, as we have said, a contribution of great value both to physiology and medicine.

To accomplish his end, new instruments of precision were required, and fortunately Dr. Oliver possessed the mechanical ingenuity to invent them. He has given us, described in this book, a new hæmoglobinometer and hæmocytometer, based on colorimetric methods—they appear to be simple of application and accurate in results; and also a hæmodynamometer or blood-pressure gauge, and an arteriometer, which is an improved form of the one previously invented by him. It is claimed for these instruments, apparently with ample justification, that they are simple, easy to work with, do not occupy much time, give accurate results, and substitute measured results for vague estimates, thus helping to precision in clinical work. A full description and mode of using these instruments is given, and also the results which the author has obtained from their employment in clinical practice. These are of great interest and of practical importance, and we think that medical men who desire to increase the accuracy of their work will be glad to adopt them. However this may be, the author's book is a real and original addition to the clinical study of the circulation, and will well repay careful study.

